# Measurement of Phase Difference for Micromachined Gyros Driven by Rotating Aircraft

**DOI:** 10.3390/s130811051

**Published:** 2013-08-21

**Authors:** Zengping Zhang, Fuxue Zhang, Wei Zhang

**Affiliations:** 1 School of Electronic Engineering, Beijing University of Posts and Telecommunications, Beijing 100876, China; E-Mail: zhangzp@bupt.edu.cn; 2 Sensing Technique Research Center, Beijing Information Science and Technology University, Beijing 100101, China; E-Mail: way_zh@163.com

**Keywords:** micromachined gyro, silicon pendulum, phase difference, rotating aircraft

## Abstract

This paper presents an approach for realizing a phase difference measurement of a new gyro. A silicon micromachined gyro was mounted on rotating aircraft for aircraft attitude control. Aircraft spin drives the silicon pendulum of a gyro rotating at a high speed so that it can sense the transverse angular velocity of the rotating aircraft based on the gyroscopic precession principle when the aircraft has transverse rotation. In applications of the rotating aircraft single channel control system, such as damping in the attitude stabilization loop, the gyro signal must be kept in sync with the control signal. Therefore, the phase difference between both signals needs to be measured accurately. Considering that phase difference is mainly produced by both the micromachined part and the signal conditioning circuit, a mathematical model has been established and analyzed to determine the gyro's phase frequency characteristics. On the basis of theoretical analysis, a dynamic simulation has been done for a case where the spin frequency is 15 Hz. Experimental results with the proposed measurement method applied to a silicon micromachined gyro driven by a rotating aircraft demonstrate that it is effective in practical applications. Measured curve and numerical analysis of phase frequency characteristic are in accordance, and the error between measurement and simulation is only 5.3%.

## 1. Introduction

**Figure 1.**
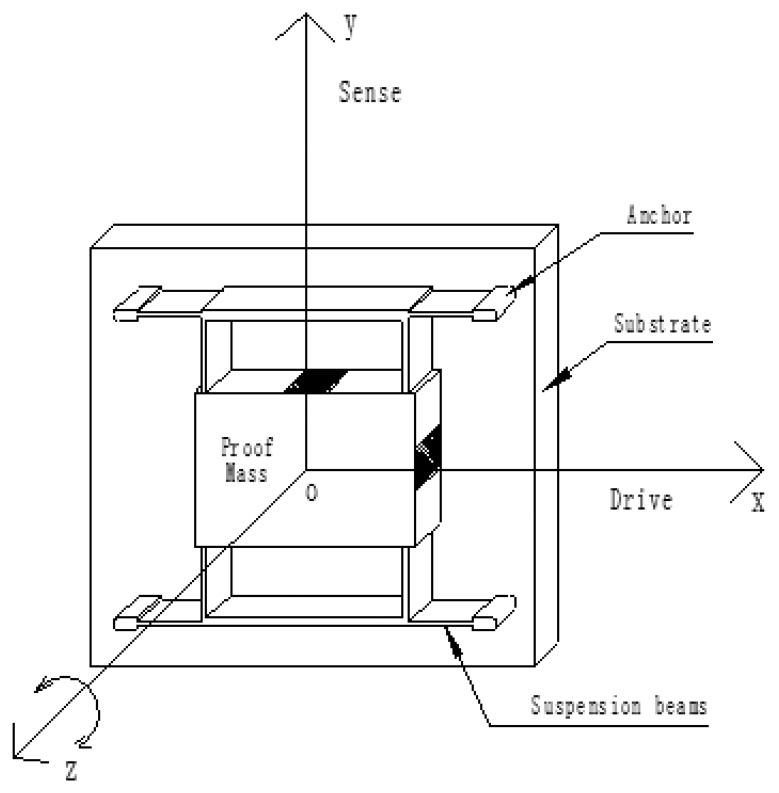
A generic MEMS gyroscope with the driving part.

With the increasing development of MEMS and inertial guidance technology, all kinds of micromachined gyros have successfully developed and are gaining increasing popularity for shared use in military and civil applications [1–3]. According to the driving structure, a MEMS gyro can be divided into two types. One is a gyro with a driving structure, and another is gyros without driving structures. The vast majority of reported micromachined rate gyroscopes utilize a vibratory proof mass suspended by flexible beams above a substrate. The primary working principle is to form a vibratory drive oscillator, coupled to an orthogonal sense accelerometer by the Coriolis force. Using anchor, the proof mass is suspended above the substrate, making the mass free to oscillate in two orthogonal directions-the drive and the sense directions (Figure 1) [4–8].

This kind of gyro is difficult to design and manufacture and the cost is high. In order to avoid the difficulties brought about by the driving part, this paper puts forward a novel gyro that uses the rotation of the aircraft itself as a driving part. In this paper, the studied gyro, which has been fabricated on s monocrystalline silicon wafer by means of bulk micromachining manufacturability techniques, belongs to the gyro without driving structure class. Since there is no driving structure, the structure is simple and easy to process [9,10]. This new gyro is used on rotating aircraft for flight attitude control. The gyro sensing element is called a silicon pendulum. Aircraft spin provides angular momentum for the silicon pendulum so that it can sense the transverse angular velocity when aircraft appears to turn. Using detection circuits, the angular vibration of the silicon pendulum is transformed into an alternating electric signal. The signal frequency is same as the spin frequency of aircraft, and the signal envelope is proportional to the transverse angular velocity.

**Figure 2.**
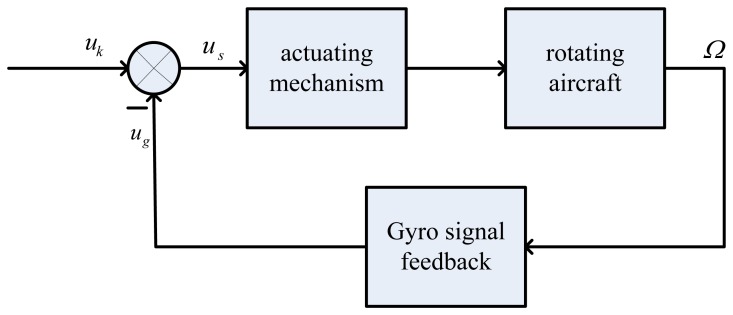
The block diagram of damping loop.

In the practical applications, dynamic characteristics of micromechanical gyroscope is the key [11–17]. For a single channel control of aircraft, the novel gyro is for damping in the whole stable loop, as shown in Figure 2. In Figure 2, *u_k_* is the control signal and *u_g_* is the gyro's output signal. The term *u_s_* is the control signal of the steering engine and it equals the value of *u_k_* minus *u_g_*. The term *u_s_* controls the aircraft rotation via the actuating mechanism and the gyro senses a transverse angular velocity of *Ω*, then the gyro outputs a signal *u_g_* and feeds it back to the end of the control signal, so a stable control loop can be obtained based on the novel gyro in the single channel control system of the rotating aircraft. The measurement object of the gyro is the input transverse angular velocity of the rotating aircraft, where *u_g_* is the gyro's output signal such that *u_g_* = *KΩcos(φ̇* − *β)*, where *K* is scale factor, *Ω* and *φ̇* are respectively the input angular velocity and spin angular velocity of the aircraft, and *β* is the phase difference between *u_k_* and *u_g_*.

Because the gyro signal is a negative feedback, the gyro signal and control signal need to be in phase. Therefore, it is a key that the phase difference be accurately measured so as to adjust and compensate the phase in practical applications.

## 2. Kinematic Equation

### 2.1. Kinematic Equation

**Figure 3.**
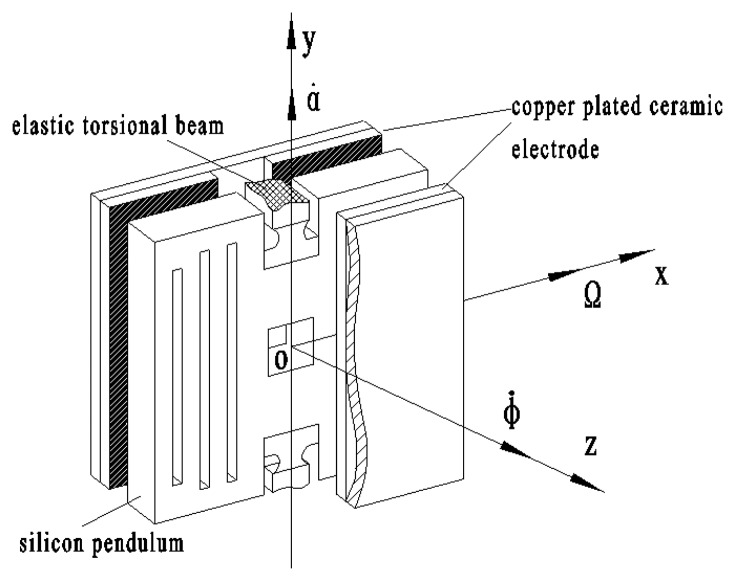
Gyro structure schematic diagram.

**Figure 4.**
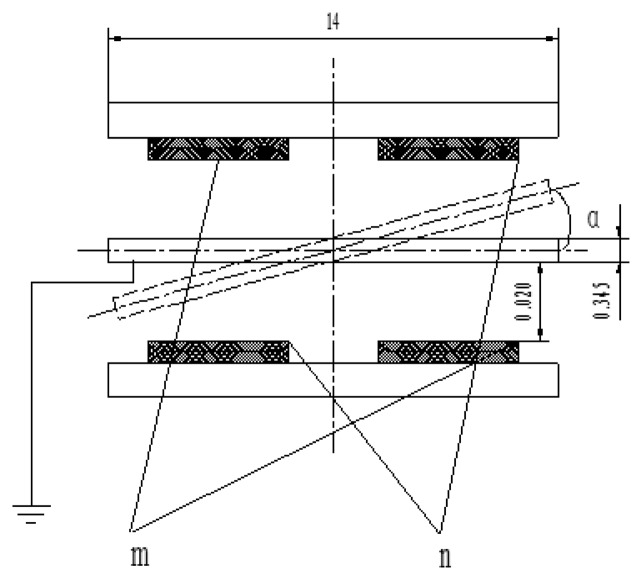
Detection capacitances.

Figure 3 shows the gyro principle structure diagram, where the middle section is a sensitive element, called a silicon pendulum. Through bulk micro-mechanical processing technology, it is fabricated on a mono-Si wafer. There are two copper plated ceramic electrodea on both sides of the silicon pendulum, on which copper has been plated by mean of a sputtering process. The plate electrode and silicon pendulum form symmetrical detection capacitances, where m is a detection capacitance positive pole and n is another detection capacitance positive pole, as shown in Figure 4.

Figure 3 shows that elastic torsion beams are located on both ends of the constraint center of the silicon pendulum and they are fixed on the frame. Nitrogen is encapsulated between the electrode and the silicon pendulum. When the silicon pendulum is vibrating around the constraint center, the capacitance changes periodically. Then, by picking up the capacitance circuit, the gyro outputs a sensitive signal.

The rotating aircraft-driven silicon micromachined gyro is installed on the rotating aircraft. In Figure 3, *oy* is the output axis, called also precession axis, *ox* is the input axis and *oz* is the driving axis, which is accordance with the spin axes of a rotating aircraft. When it is rotating at a high speed around the spin axis, the aircraft will drive the silicon pendulum rotating at the same angular velocity as the aircraft spin angular velocity *φ̇* and the silicon pendulum will also acquire angular momentum.

**Figure 5.**
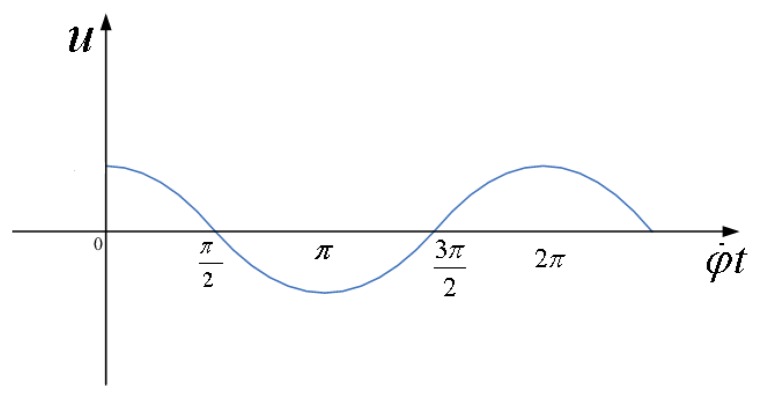
Output voltage waveform with a constant input angular velocity.

**Figure 6.**
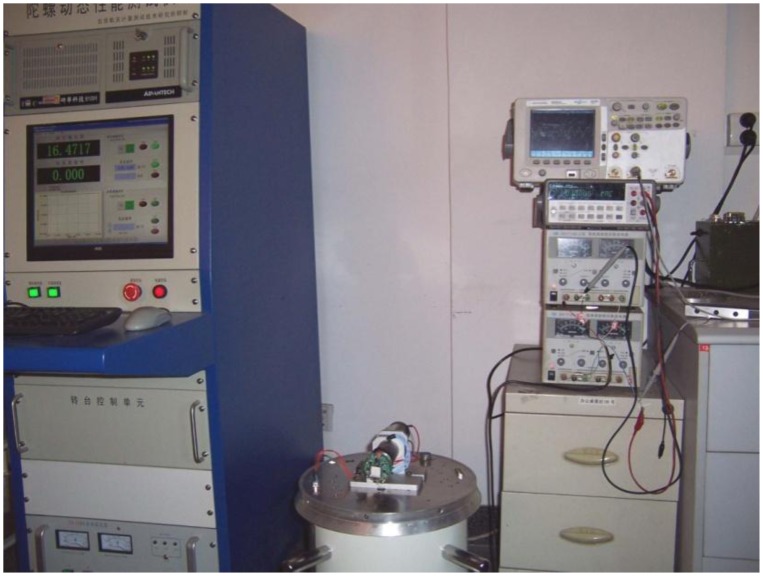
Experimental platform.

Angular momentum is same in the direction as the *oz* axis. If the aircraft turns with angular velocity of *Ω* around the input axis, the silicon pendulum will be forced to produce precession. Thus, along with the direction of precession *oy* axis, the precession moment acts upon silicon pendulum. The precession moment can be balanced with various moments such as the inertia moment, damping torque and elastic moment. Concerning these moments, because the silicon pendulum is vibrating around the precession axis, the angular acceleration generates inertia moment. Having angular velocity, the squeeze film resistance of nitrogen generates a damping torque, and the elastic torsion beam also generates elastic moment. In short, when aircraft undertakes a forced turn around the input axis *ox*, as a result, the silicon pendulum will generate precession to make angular momentum tend to the input transverse angular velocity *Ω*. The term *α̇* represents the angular velocity of the silicon pendulum vibration. Suppose that the input angular velocity *Ω* is a constant, with the aircraft spin, the silicon pendulum will generate a simple harmonic vibration. After the detection circuit and conditioning circuit, the gyro will output a periodic voltage signal, as shown in Figure 5. A case is introduced to validate the correctness and applicability of the Si micromachined gyro driven by a rotating aircraft. The experimental platform is shown in Figure 6.

**Figure 7.**
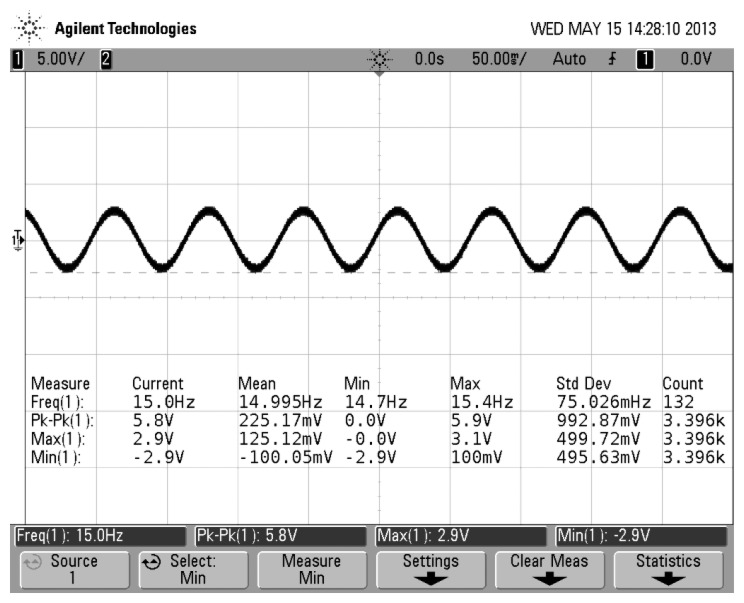
Experimental waveform.

**Figure 8.**
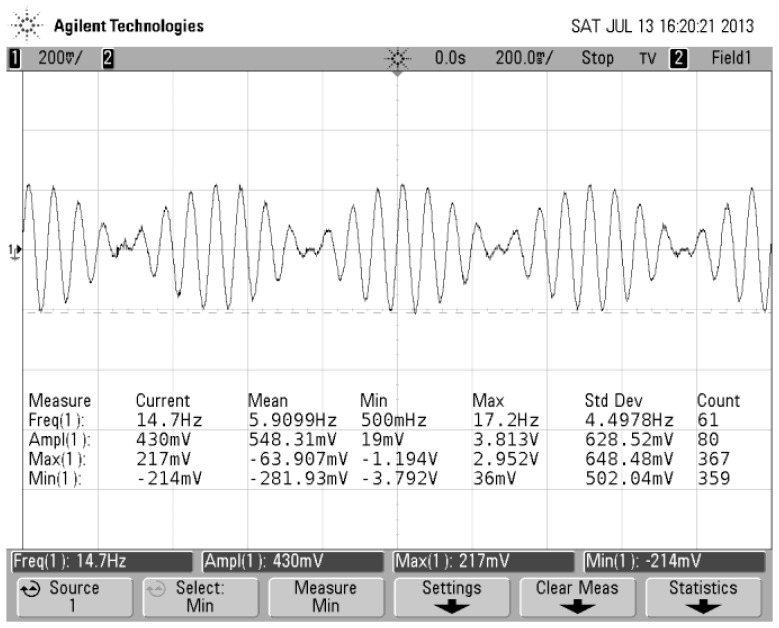
AM waveform.

**Figure 9.**
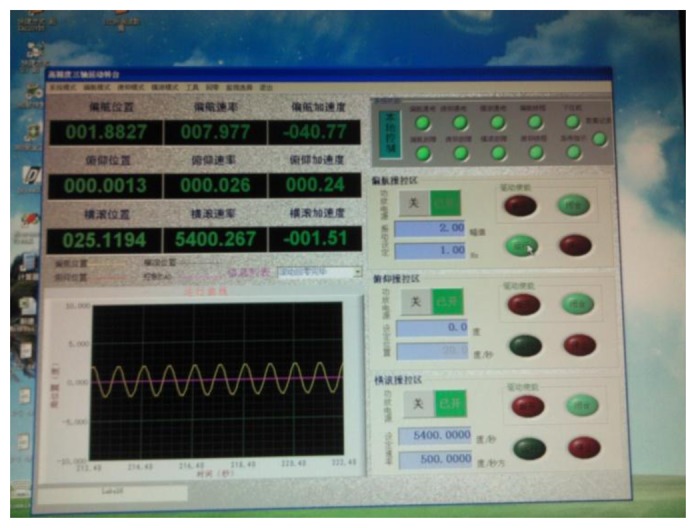
Test control panel.

Figure 6 shows that the gyro was installed on an emulator and emulator was mounted on an angular vibration table. The spin angular velocity was given by the emulator and the value of the spin angular velocity had been adjusted to 5,400°/s (15 Hz). Input transverse angular velocity was provided by the angular vibration table and was inputted a constant value of 180°/s. The gyro's output signal was measured via a scope and the measured waveform is shown in Figure 7. If the aircraft is not rotating at a constant rate but rather a sinusoidal function with a frequency of 1 Hz, the gyro's output signal is a AM signal and the envelope of the signal corresponds to the sinusoidal function, as shown in Figure 8. The test control panel is shown in Figure 9.

Waveforms in Figure 5 and Figure 7 are perfectly in accordance. Therefore, testing validates that the previous analysis is correct. With respecting to the above-mentioned motion, it can be illustrated by rigid motion around fixed point. Therefore, using the Euler dynamic equation based on rigid movement around a fixed point and its projection on the procession axis, we will easily derive the motion equation to express Equation (1) as in [10]

(1) $$J_{y}\ddot{\alpha }+D\dot{\alpha }+\left[\left(J_{z}-J_{x}\right){\dot{\varphi }}^{2}+K_{T}\right]\alpha =\left(J_{z}+J_{y}-J_{x}\right)\Omega \dot{\varphi }\cos\left(\dot{\varphi }t\right)$$

where *J_x_*, *J_y_* and *J_z_* represent the moment of inertia of the silicon pendulum relative to *x*, *y* and *z*, respectively. *K_T_* is the torsion stiffness of the elastic torsion beam and *D* is a damping factor. *α̇* represents the value of the vibration angular velocity of the silicon pendulum, *Ω* represents the value of the input angular velocity, and *φ̇* represents the value of the spin angular velocity of the aircraft. To simplify Equation (1) further, it can be written as:

(2) $$\ddot{\alpha }+2\varsigma \omega_{n}\dot{\alpha }+{\omega_{n}}^{2}\alpha =f_{0}\cos\left(\dot{\varphi }t\right)$$

where:

(3) $$\begin{array}{c} {\omega_{n}}^{2}=\frac{1}{J_{y}}\left[\left(J_{z}-J_{x}\right){\dot{\varphi }}^{2}+K_{T}\right]\\ \varsigma =\frac{D}{2\omega_{n}J_{y}}=\frac{D}{2\sqrt{J_{y}\left[\left(J_{z}-J_{x}\right){\dot{\varphi }}^{2}+K_{T}\right]}}\\ f_{0}=\frac{1}{J_{y}}\left(J_{z}+J_{y}-J_{x}\right)\Omega \dot{\varphi } \end{array}$$

Equation (2) is a second order constant coefficient linear differential equation, then, the solution of Equation (2) is given by:

(4) $$\alpha =A_{1}e^{-nt}\cos\left(\omega_{f}t+\delta \right)+A_{2}\cos\left(\dot{\varphi }t-\beta \right)$$

where *A*_1_ is a integration constant, which value is determined by the initial conditions, *n* is an attenuation factor, including *n*=*ςω_n_* and $\omega_{f}=\sqrt{{\omega_{n}}^{2}-n^{2}}$. Equation (4) indicates that the solution consists of a damping vibration and a harmonic vibration. The first item of Equation (4) would be quickly attenuated to zero, then the forced vibration will reach steady state and it can be calculated as follows:

(5) $$\alpha =\frac{\left(J_{z}+J_{y}-J_{x}\right)\dot{\varphi }\Omega }{\sqrt{{\left[\left(J_{z}-J_{x}-J_{y}\right){\dot{\varphi }}^{2}+K_{T}\right]}^{2}+{\left(D\dot{\varphi }\right)}^{2}}}\cos\left(\dot{\varphi }t-\beta \right)$$

Then, the amplitude of the angular vibration can be written as:

(6) $$\alpha_{m}=\frac{\left(J_{z}+J_{y}-J_{x}\right)\dot{\varphi }}{\sqrt{{\left[\left(J_{z}-J_{x}-J_{y}\right){\dot{\varphi }}^{2}+K_{T}\right]}^{2}+{\left(D\dot{\varphi }\right)}^{2}}}\Omega$$

The phase difference is:

(7) $$\beta =\arctan\left(\frac{2n\dot{\varphi }}{{\omega_{n}}^{2}-{\dot{\varphi }}^{2}}\right)$$

### 2.2. Kinetic Parameters

**Figure 10.**
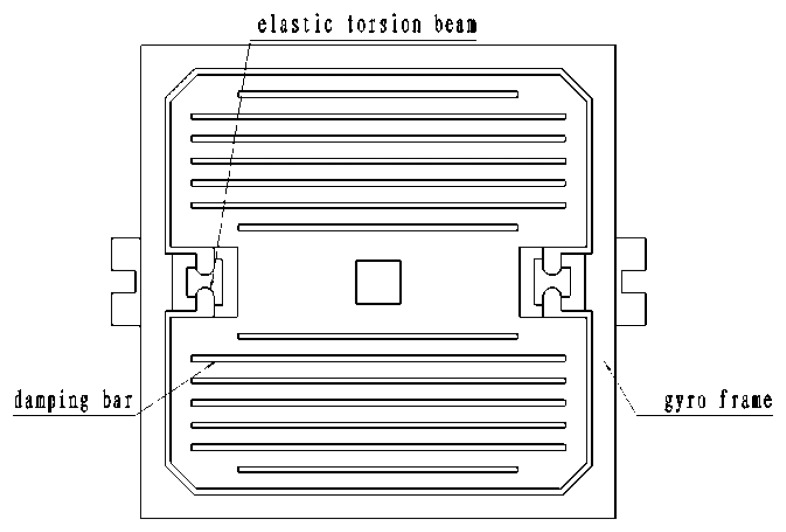
Silicon pendulum structure.

Figure 10 is the structural diagram of the silicon pendulum. The gyro frame is fixed during packaging and cannot move. There is a movable part, called the silicon pendulum, inside the frame. Its two ends are elastic torsion beams, which connect with the frame. There are 14 damping bars on two sides of the silicon pendulum. Squeeze film damping, has a big impact on the dynamic response of MEMS microstructure, and can be used to adjust the quality factor of the micro-mechanical structure. Increased damping bars can reduce the pressure of the gas film, and the gas film damping is reduced accordingly, so in the MEMS design, in order to speed up the release or decrease damping, we usually include a release bar or so-called damping bar in the silicon pendulum.

**Figure11:**
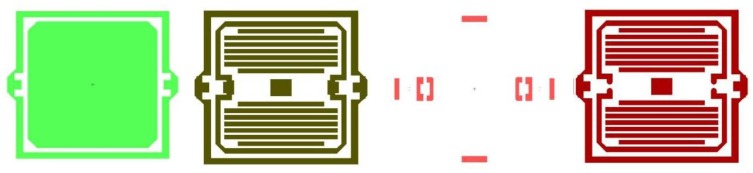
The masks of four steps.

In the chip process, we have four steps. The first step shapes a movable section, the second step etches the damping bars, the next is buffer layer etching on the beams and the last step forms the silicon pendulum. The masks of the four steps and chip are shown in Figure 11.

**Figure 12.**
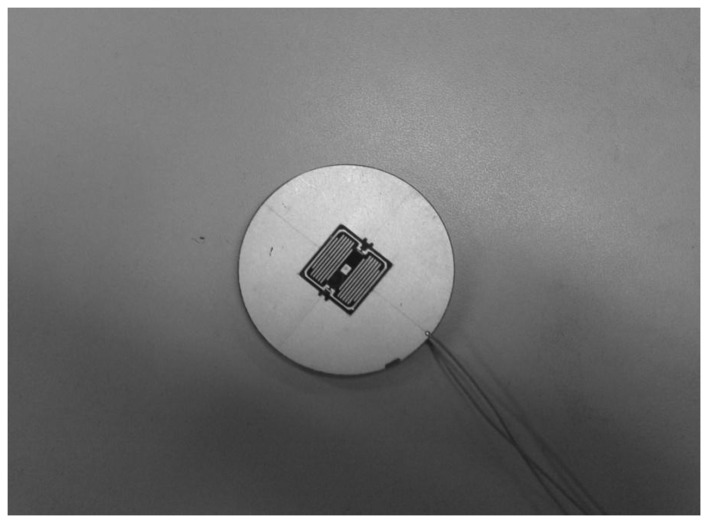
Picture of the chip.

Using the above four step etching, the final chip is obtained. A picture of the chip is shown in Figure 12.

#### 2.2.1. Torsion Stiffness

Considering that it is convenient to calculate the torsion stiffness of an elastic torsion beam, it is supposed that on processing the torsion angle is proportional to the length of the beam, warping of the cross sections of elastic torsion beam are same, and values are same, but in opposite direction, for the torsion moment of the two ends of an elastic torsion beam.

Under the condition of the above assumptions, using elastic mechanics, the torsion stiffness with rectangular cross section can be obtained by:

(8) $$K_{T}=\frac{512Gt^{3}w}{\pi^{4}L}\sum_{n=1,3,5,...}^{\infty }\frac{1}{n^{4}}\left(1-\frac{2t}{n\pi w}\tanh\frac{n\pi w}{2t}\right)$$

where *L*, *w*, and *t* are the length, width and thickness of the elastic torsion beam, respectively. *G* is the sheer modulus of the silicon material.

Practically the torsion angle *α* is limited to 0.003 rad, *K_T_* is large enough, thus we can simplify Equation (8), and then Equation (9) can be obtained:

(9) $$K_{T}=\frac{2}{3}\cdot \frac{Gt^{3}w}{L}$$

If *G*, *L*, *w*, and *t* are chosen to be:

(10) $$L=600\mu \text{m},w=400\mu \text{m},t=75\mu \text{m},G=5.1\times 10^{10}{\text{N}/\text{m}}^{2}$$

the calculated result is:

(11) $$K_{T}=2.507\times 10^{-3}\text{N}\cdot \text{m}$$

#### 2.2.2. Damping Factor

The displacement of the silicon pendulum edge node is far less than both the lateral size and gap relative to the electrodes in the torsion motion of Figure 4. By using numerical FEA simulation, the maximum displacement is only 886 nm. Therefore, in the analysis of the problem, we make the assumption that the silicon pendulum undergoes a rigid motion of small amplitude, the gas film gap is just a time function, and the linear solution of the Reynolds equation is given for calculating the damping coefficient of the silicon pendulum.

When a rectangular plate with length *A* and width *B* is moving relative to the undersurface of a gap distance of *h*, the damping coefficient of the squeeze film resistance can be expressed by:

(12) $$f=\frac{F_{\text{damp}}}{dh/dt}=\frac{AB^{3}\mu }{h^{3}}\left[1-\frac{192}{A\pi^{5}}\sum_{n=1,3,5,...}^{\infty }\frac{1}{n^{5}}\tan h\frac{n\pi^{4}}{2B}\right]$$

where *μ* is the gas viscosity coefficient.

Since the infinite series can quickly converge, the damping coefficient is approximately equal to the first term of Equation (12), that is:

(13) $$f=\frac{AB^{3}\mu }{h^{3}}\left[1-\frac{192}{A\pi^{5}}\tanh\frac{\pi^{4}}{2B}\right]$$

In order to calculate the damping factor *D* shown in Figure 10, the first step is to figure out the damping factor of the entire silicon pendulum, then, to calculate the damping factor of the damping hole, notch and fourteen damping bars. Finally, the entire silicon pendulum damping factor minus the damping factors of the missing parts above. As a result, the real damping factor of the silicon pendulum is obtained.

The chosen relevant dimensions are: *h* = *20* μm, *A* = *B* =*14* mm, *μ* = *17.81×10*^−^*^6^* P_a_·s, thus, the damping factor of the silicon pendulum can be figured out:

(14) $$D=1.676\times 10^{-4}\text{N}\cdot \text{m}\cdot \text{s}$$

#### 2.2.3. Moment of Inertia

Moment of inertia (*J_x_*, *J_y_*, *J_z_*) relative to the principal axes of inertia are calculated in the same way as the damping factor. Finally, the obtained moment of inertia can respectively be written as:

(15) $$\begin{array}{c} J_{x}=2.27794\text{g}\cdot {\text{mm}}^{2}\\ J_{y}=2.1527\text{g}\cdot {\text{mm}}^{2}\\ J_{z}=4.43064\text{g}\cdot {\text{mm}}^{2} \end{array}$$

## 3. Phase-Frequency Characteristics

### 3.1. Phase-Frequency Characteristics

In order to analyze the phase-frequency characteristics of the Si micromachined gyro driven by a rotating aircraft, the vibration of the silicon pendulum can be represented as a SISO system, of which the excitation signal is given by *x*(*t*) and the response signal is given by *y*(*t*). The term *x*(*t*) will simulate the excitation generated by the moment of procession. The term *y*(*t*) simulates the displacement of the angular vibration of the silicon pendulum. Thus, Equation (2) can be expressed in another form as follows:

(16) $$\frac{d^{2}y}{dt^{2}}+2\varsigma \omega_{n}\frac{dy}{dt}+{\omega_{n}}^{2}y=x\left(t\right)$$

Using the Laplace transformation, the transfer function of Equation (16) can be written as:

(17) $$H\left(s\right)=\frac{1}{s^{2}+2\varsigma \omega_{n}s+{\omega_{n}}^{2}}$$

Then, the frequency characteristic is:

(18) $$H\left(j\omega \right)=\frac{\left(\frac{1}{{\omega_{n}}^{2}}\right)}{1-{\left(\frac{\omega }{\omega_{n}}\right)}^{2}+j2\varsigma \left(\frac{\omega }{\omega_{n}}\right)}$$

Finally, we can easily obtain the phase-frequency characteristic:

(19) $$\varphi \left(\omega \right)=\arctan\frac{2\varsigma \left(\frac{\omega }{\omega_{n}}\right)}{{\left(\frac{\omega }{\omega_{n}}\right)}^{2}-1}$$

In actual applications, the working frequency (ω) is less than the natural frequency of *ω_n_*, so Equation (19) shows that the output signal has a phase lag relative to the input, or said differently, the silicon pendulum angular vibration and input angular velocity have a phase delay. When the spin angular velocity of the aircraft reaches the natural frequency of the silicon pendulum, this phase lag is 90 degrees.

### 3.2. Phase-Frequency Characteristics of Different Damping Ratios

**Figure 13.**
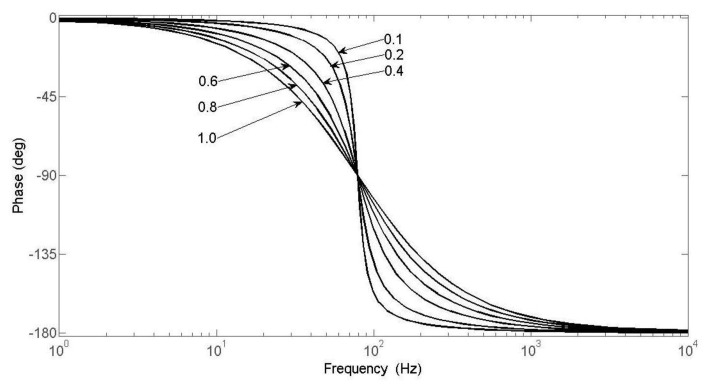
Phase frequency characteristic curves for different damping ratios.

The phase-frequency characteristics are simulated and plotted for a Si micromachined gyro driven by a rotating aircraft. The plotted curve is shown in Figure 13.

In Figure 13, it is clearly shown that when the damping ratio is smaller, the phase difference also becomes less. Practically we can always design to an appropriate damping ratio, such as 0.5 or 0.7.

### 3.3. Conditioning Circuits Impact

**Figure 14.**
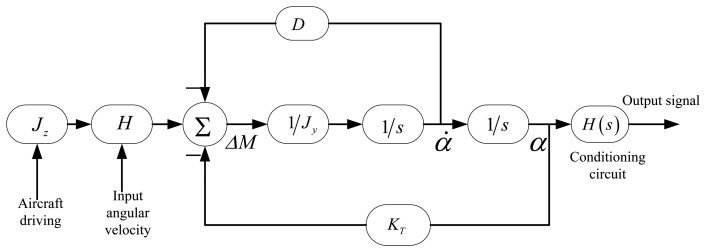
Block diagram of gyro working principle.

The Si micromachined gyro principle block diagram is shown in Figure 14, which shows that the phase difference is not only relevant to the vibration of the silicon pendulum, but also affected by the conditioning circuits. Phase difference is equal to the sum of the phase difference of two parts. The sconditioning circuits consist of a differential amplifier circuit, band-pass filter circuit, low-pass filter circuit and gain circuit.

We chose an AD620 chip, which constitutes the differential amplifier circuit. We connect the gyro's sensed signal with its noninverting and inverting inputs. Its magnification times are adjusted by a resistor cross input. Meanwhile, we add a LPF at the noninverting and inverting inputs, respectively. The transfer function of the differential amplifier circuit is written as:

(20) $$H_{1}\left(s\right)=\frac{25.7}{10^{-5}s+1}$$

Because the gyro's working frequency ranges from 1 Hz to 50 Hz, the band width of the BPF is designed to be 81 Hz. The transfer function for BPF is:

(21) $$H_{2}\left(s\right)=\frac{0.64s^{2}+3520s}{0.64s^{2}+324s+2000}$$

In order to filter the noise, we design the second order LPF. Its cut-off frequency is 63.5 Hz and transfer function is:

(22) $$H_{3}\left(s\right)=\frac{1}{6.618\times 10^{-6}s^{2}+3.52\times 10^{-3}s+1}$$

For the gain circuit, its function is mainly amplifying the signal and large transmission bands. The peak gain is 17.3 dB, so the transfer function is designed to be:

(23) $$H_{4}\left(s\right)=\frac{118.252s+68000}{4.96\times 10^{-4}s^{2}+16.213s+22000}$$

Then, for the entire conditioning circuits, the transfer function is:

(24) $$H\left(s\right)=H_{1}\left(s\right)H_{2}\left(s\right)H_{3}\left(s\right)H_{4}\left(s\right)$$

**Figure 15.**
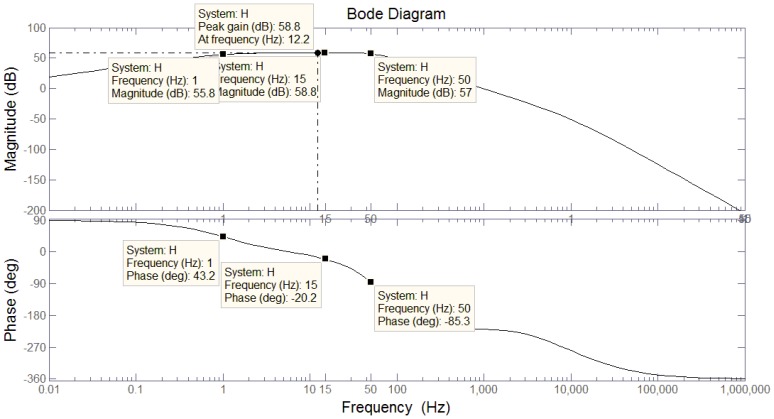
Bode plot of Equation (24).

According to Equation (24), we solve the amplitude-frequency characteristic and phase frequency characteristic. The Bode plot is as shown in Figure 15.

According to the amplitude-frequency characteristics of Figure 15, in the range of 1∼50 Hz, the gain is relatively flat. In addition, the phase frequency characteristics of Figure 15 show that the phase difference curve is approximately linear.

## 4. Dynamic Simulation of Phase Difference

### 4.1. Simulation Modeling

**Figure 16.**
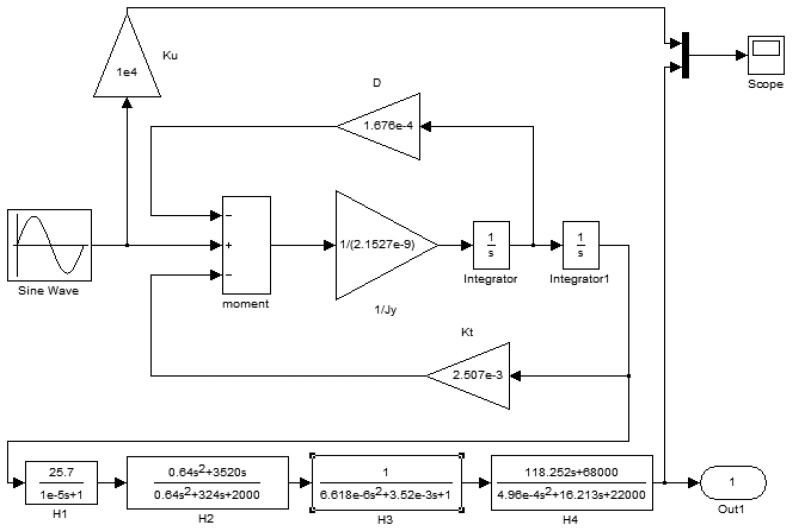
Modeling of the dynamic simulation.

The model structure built according to the working principles of the gyro is shown in Figure 16. It includes the second order vibration model of a silicon pendulum and the conditioning circuit model.

In Figure 16, an input periodic signal simulates the excitation of the second-order vibration system. The frequency of the periodic signal is the spin frequency of the aircraft. The constant transverse angular velocity is modulated on the sinusoidal wave amplitude. The second order vibration system is a closed loop system. Due to the vibration angular velocity of the silicon pendulum, the damping moment, which is formed by the damping factor *D*, feeds back to the input. Meanwhile, the torsion moment, which is formed by the torsion stiffness *K_T_*, also feeds back to the input. To summarize, the precession moment, damping moment and torsion moment together form a loop with negative feedback. There are two differential operators *S* in the loop model. The conditioning circuit models are located on the last step of the modeling.

### 4.2. Dynamic Simulation

**Figure 17.**
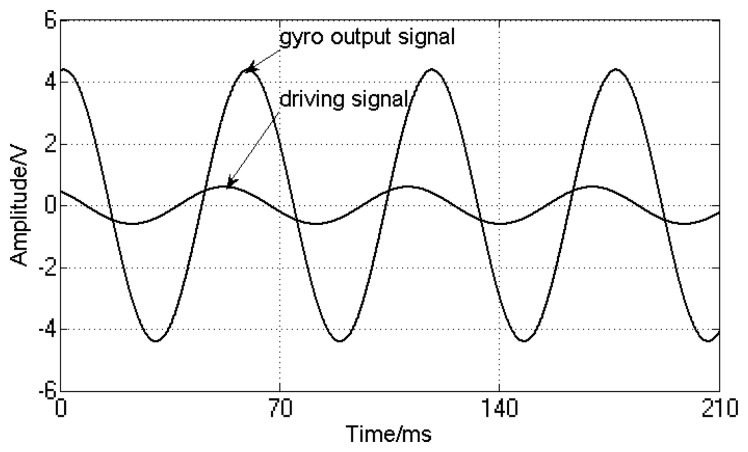
Dynamic simulation waveform.

The driving signal frequency is set to 15 Hz and simulates the aircraft spin frequency. Because the input drive signal simulates the aircraft rotation driving, when the aircraft has a transverse angular velocity, the silicon pendulum will generate an angular vibration. Because the silicon pendulum is of microscopic scale, the moment of inertia around the driving axis is very small, so the angle of vibration is also very small and the detected signal is a small signal. Therefore, the input driving signal amplitude is smaller in the modeling. In order to compare the phase difference between the input driving signal and the gyro output signal, a gain of *K_u_* between the driving signal and the oscilloscope is added for phase difference comparison. Using dynamic simulation, the simulation results are shown in Figure 17.

Figure 17 indicates that the gyro output frequency is same as the driving signal and their frequencies are 15 Hz. The phase delay is 8 ms, that is, the phase lag is:

(25) $$\Delta \varphi =2\pi f\Delta t\frac{180}{\pi }$$

where *f* is the spin frequency of the aircraft, Δ*t* is the phase delay. If *F* = *15* Hz, *Δt* = *8* ms are chosen then the calculated phase difference is *Δφ* = *43.2*°.

## 5. Measuring of Phase Difference

### 5.1. Measuring Platform

**Figure 18.**
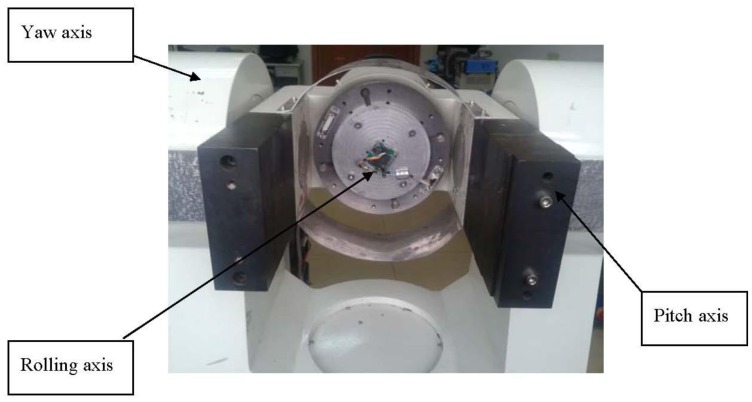
Measuring platform.

**Figure 19.**
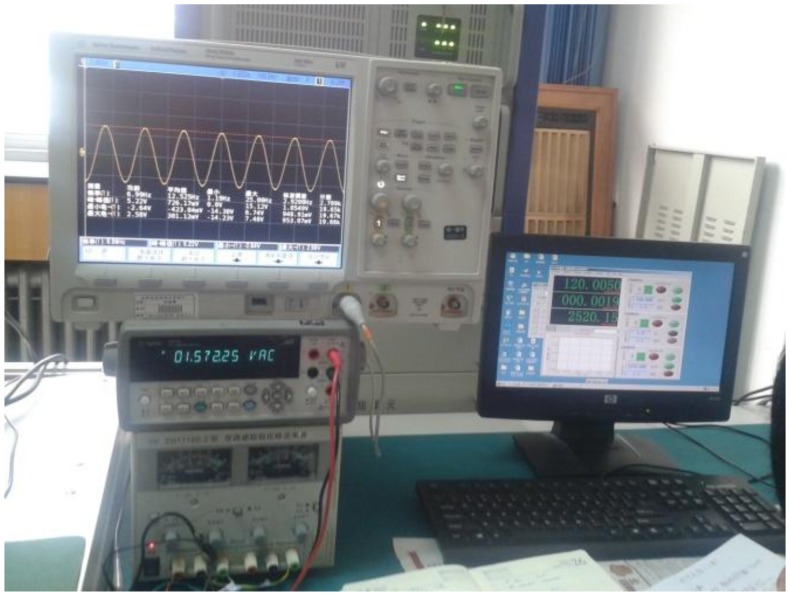
Control platform.

**Figure 20.**
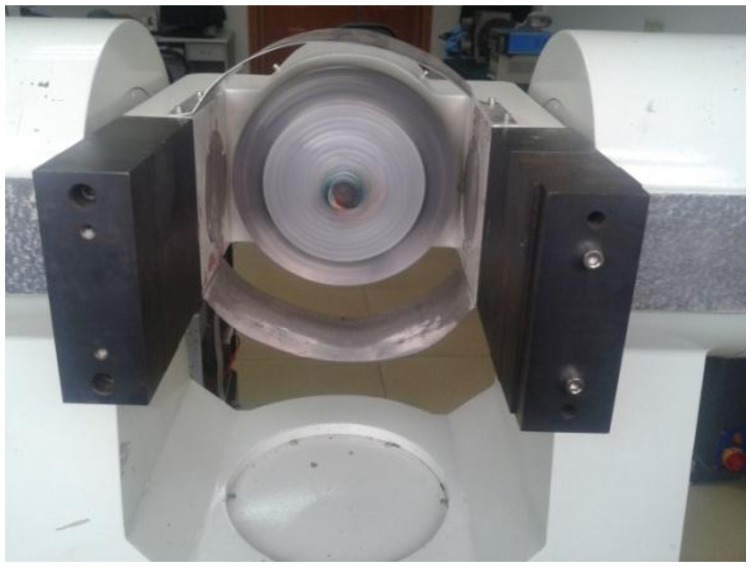
Working status.

The gyro was installed on a MEMS three-axis precision turntable. Figure 18 shows the installation on the turntable. Figure 19 is the control table, and Figure 20 shows the working status.

Figure 20 shows that the rolling axis was rotating at a certain speed and generated angular momentum for the silicon pendulum. When the input transverse angular velocity was generated by the yaw axis or pitch axis, the gyro senses an input transverse angular velocity. In order to measure the phase difference with respect to different frequencies, we adjust different rolling velocities via the control platform in Figure 19. Then we compare the phase between the gyro's output signal and angular displacement of the rolling axis, and we obtain the phase difference with respect to different frequencies.

### 5.2. Measurements

Given an input angular velocity of 180°/s, the turntable rolling axis is rotating at a high speed. At the same time, through the data acquisition card of the MEMS three-axis precision turntable, we will obtain data of the frame angular displacement and gyro output signal. After data acquisition is completed, the speed of the rolling axis is increased. In the same way, measurement is accomplished.

**Figure 21.**
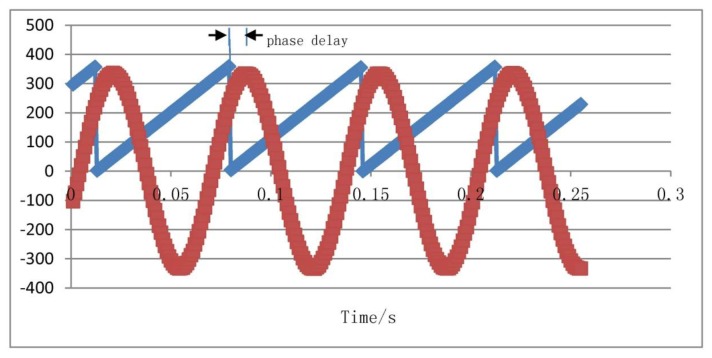
Phase delay comparison chart.

In order to compare to the simulation result as shown in Figure 17, we choose sampled data of *φ* = *15* Hz, *Ω* = *180*°/s. Using these sampled data, we plot a curve, as shown in Figure 21. The blue line represents the turntable rolling axis angular displacement, while the red one represents the gyro output signal.

**Figure 22.**
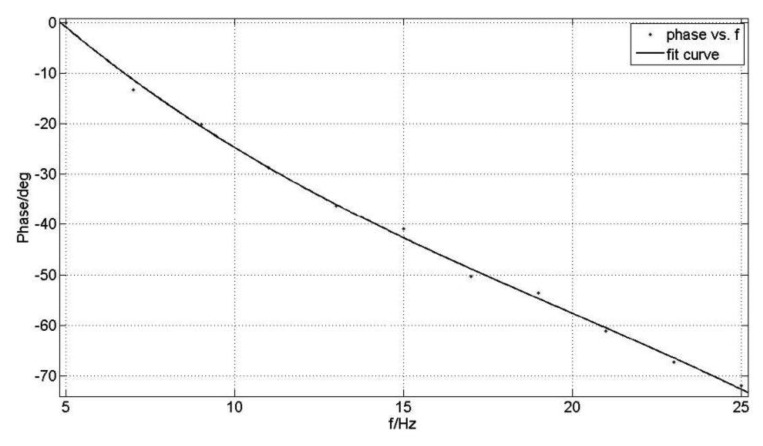
The experimental phase-frequency characteristic curve.

As shown in the Figure 21, the phase difference between the gyro output signal and driving reaches a value of 40.9°. Under the different rolling frequency, the calculated phase differences are recorded in Table 1. The fitting curve of the measured phase frequency characteristic is shown in Figure 22.

## 6. Results and Discussion

Figure 22 shows that the characteristic phase frequency curve, ranging from 5 Hz to 25 Hz, is in accordance with the simulation curve in Figure 13. Therefore, the results demonstrate that the measurement method is correct, but because of the maximum limits of the rolling frequency of the turntable rolling axis, the range of measured frequency is not outside the limit of 30 Hz. For dynamic measurements of a spin frequency of 15 Hz, the simulation result of the phase difference is 43.2° and the measured value is 40.9°. The error is only 5.3%, so the measured phase difference can meet the needs of adjusting the phase in single channel control applications of rotating aircraft.

## 7. Conclusions

This paper introduces a new type of micromachined gyro, and its working principles were analyzed. For the MEMS micromachined gyro driven by a rotating aircraft, the movement equation is presented and the dynamic parameters are calculated. Based on the motion equation, we analyzed the phase frequency characteristics and plotted the characteristic phase frequency curve under different damping ratio scenarios. Combined with the dynamic parameters, a dynamic simulation model has been set up. When the spin frequency is 15 Hz, the obtained phase difference simulation is 43.2°. On the basis of this analysis, we designes a phase delay measurement method. Using this method, we have measured the characteristic phase frequency curve. Ranging from 5 Hz to 25 Hz, the measured curve was in accord with the simulation. When the spin frequency is chosen as 15 Hz, by measurements we obtained a phase difference of 40.9°. The relative error with respect to the simulation is only 5.3%. The measured results prove that the accuracy is good and the data of the measured phase difference can meet the requirements of adjusting the phase for the single channel control of a rotating aircraft.

## Figures and Tables

**Table 1.** Phase-frequency characteristic experimental records.

*f/Hz*	5	7	9	11	13	15	17	19	21	23	25
*φ/deg*	0	−13.3	−20.1	−28.8	−36.3	−40.9	−50.3	−53.6	−61.2	−67.3	−72
